# Galectin-1 correlates with inflammatory markers and T regulatory cells in children with type 1 diabetes and/or celiac disease

**DOI:** 10.1093/cei/uxad131

**Published:** 2023-12-13

**Authors:** Emanuel Fryk, Åsa Wilsson, Andrea Tompa, Per-Anders Jansson, Maria Faresjö

**Affiliations:** Wallenberg Laboratory, Department of Molecular and Clinical Medicine, Institute of Medicine, University of Gothenburg, Gothenburg, Sweden; Department of Natural Science and Biomedicine, School of Health and Welfare, Jönköping University, Jönköping, Sweden; Department of Natural Science and Biomedicine, School of Health and Welfare, Jönköping University, Jönköping, Sweden; Division of Medical Diagnostics, Department of Laboratory Medicine, Region Jönköping County, Sweden; Wallenberg Laboratory, Department of Molecular and Clinical Medicine, Institute of Medicine, University of Gothenburg, Gothenburg, Sweden; Department of Life Sciences, Division of Systems and Synthetic Biology, Chalmers University of Technology, Gothenburg, Sweden

**Keywords:** galectin-1, type 1 diabetes, celiac disease, immune markers, children

## Abstract

Type 1 diabetes (T1D) and celiac disease (CeD) are common autoimmune diseases in children where the pathophysiology is not fully characterized. The autoimmune process involves a complex scenario of both inflammatory and regulatory features. Galectin-1 (GAL-1) has a wide range of biological activities e.g. interaction with immune cells. We examined the relationship between GAL-1 and soluble immune markers and T-cell subsets in a cohort of children with T1D and/or CeD relative to healthy children. GAL-1, together with several soluble immune markers [e.g. interleukins (IL)], tumor necrosis factor (TNF), acute phase proteins, and matrix metalloproteinases (MMP) were measured in sera from children with T1D and/or CeD by fluorochrome (Luminex) technique using children without these diseases as a reference. Subgroups of T cells, including T-regulatory (Treg) cells, were analysed by flow cytometry. Association between GAL-1, pro-inflammatory markers, and Treg cells differed depending on which illness combination was present. In children with both T1D and CeD, GAL-1 correlated positively with pro-inflammatory markers (IL-1β, IL-6, and TNF-α). Composite scores increased the strength of correlation between GAL-1 and pro-inflammatory markers, Th1-associated interferon (IFN)-γ, and T1D-associated visfatin. Contrary, in children diagnosed with exclusively T1D, GAL-1 was positively correlated to CD25^hi^ and CD25^hi^CD101^+^ Treg cells. For children with only CeD, no association between GAL-1 and other immune markers was observed. In conclusion, the association observed between GAL-1, soluble immune markers, and Treg cells may indicate a role for GAL-1 in the pathophysiology of T1D and, to some extent, also in CeD.

## Teaser

Type 1 diabetes (T1D) and celiac disease (CeD) are common autoimmune diseases in children where pathophysiology is not fully characterized. The autoimmune process involves a complex scenario of both inflammatory and regulatory features. Galectin-1 (GAL-1) has a wide range of biological activities e.g. interaction with immune cells and the association observed between GAL-1, soluble immune markers, and T regulatory (Treg) cells may indicate a role for GAL-1 in the pathophysiology of children with T1D and, to some extent, also in children with CeD.

## Introduction

Type 1 diabetes (T1D) and celiac disease (CeD) are both characterized by an autoimmune feature. The prevalence of T1D is increasing globally, and the underlying pathophysiology behind β-cell failure is poorly understood [1]. There is no cure for T1D, and the only treatment available is insulin. In CeD, the autoimmune process primarily affects the small intestine, and existing treatment is focused on the complete exclusion of gluten from the diet [2]. New therapeutic approaches and alternative treatments would be beneficial, as current treatment is associated with severe physical and psychosocial burdens. To achieve this goal, we need to understand the immunological mechanisms behind these autoimmune diseases and the complexity if these two diseases arise in the same individual.

The autoimmune process observed in both T1D and CeD involves a complex scenario of different immune cells that, through activation, upregulate cell surface markers and secrete a wide range of soluble immune markers with both pro-inflammatory and regulatory properties. The autoimmune process in T1D is associated with the effector function of T helper (Th) 1 cells, which produce interferon (IFN)-γ and are key players in the destruction of the insulin-producing β cells. Th subgroups, e.g. Th17 and Th22, secreting interleukin (IL)-17 and IL-22, respectively, are also of relevance in T1D pathophysiology [3, 4]. Furthermore, the pro-inflammatory immune markers IL-1α, IL-1β, IL-2, and IL-6; tumor necrosis factor (TNF)-α/-β; and macrophage inflammatory proteins (MIP)-1α and MIP-1β are important for the autoimmune process of T1D [5, 6]. Acute-phase proteins (APP), such as procalcitonin (PCT), ferritin, tissue protein activator (tPA), fibrinogen, and serum amyloid A (SAA), may have both pro- and anti-inflammatory effects on different immune cells [7]. In both T1D and CeD, affected levels of several APPs have been reported [8, 9]. Adipocytokines play a significant role in the pathogenesis of low-grade inflammation associated with chronic inflammatory and autoimmune diseases [10, 11]. Visfatin can induce and regulate both the pro-inflammatory (IL-1β, IL-6, and TNF-α) and anti-inflammatory T regulatory (Treg)-associated cytokines (e.g. IL-10). The pro-inflammatory role of resistin in different inflammatory processes is mediated by stimulation of TNF-α, IL-1β, and IL-6 [11]. Similarly, adipocytokines are related to intestinal inflammatory diseases, which share genetics and functional pathways with CeD [12]. Matrix metalloproteinases (MMPs) are important endopeptidases involved in tissue remodeling in both physiological and pathological conditions and can promote or inhibit inflammatory processes [13, 14]. Dysregulation of MMPs has been observed in both T1D and CeD [15, 16]. Finally, Treg cells are believed to have an important role in the development of both T1D and CeD, and several cytokines have been associated with this regulation [17, 18].

Galectin-1 (GAL-1) is a β-galactoside-binding protein ubiquitously expressed in the human body [19, 20]. Important activities of galectins are initiation, amplification, and resolution of inflammatory responses, including involvement in T cell–mediated disorders, including both acute and chronic inflammation [19, 21]. In general, galectins are found in activated immune cells such as macrophages and dendritic cells, B cells, and T cells-like Treg cells [22, 23]. GAL-1 is overexpressed in Treg cells (CD4^+^CD25^+^) and treatment with an anti-GAL-1-specific antibody blocks the immunosuppressive effect and thus its ability to inhibit T-cell effector functions [24]. GAL-1 from Tregs may also exert different effects on T effector cells in animal models with or without T1D [25]. In autoimmune-prone nonobese diabetic (NOD) mice, treatment with GAL-1 reduces the amount of Th1 cells, increases the numbers of T-cells producing IL-4, IL-5, or IL-10 in response to islet antigens, and prevents the onset of hyperglycemia at both early and subclinical stages of T1D [26]. Th17 cells also secrete higher levels of GAL-1 compared to the secretion of this protein by Th1 and Th2 cells [27]. Furthermore, GAL-1 reduces immune cell infiltration in NOD mice [28]. This is evident since thymic epithelial cells express GAL-1 and trigger apoptosis of thymocytes through a GAL-1-dependent mechanism, suggesting that this lectin has functions related to central T-cell tolerance [29].

Circulating GAL-1 is associated with several diseases, including T1D and type 2 diabetes (T2D), allergic asthma, and immunity-escape of malignant tumors [30–32]. GAL-1 expression is also increased by inflammatory processes including inflammatory bowel disease, osteoarthritis, inflammation in diabetic retinopathy, and circulating CRP in a general population [33–36]. However, lower levels of GAL-1 have been observed in patients with T1D due to reduced secretion from monocytes [27]. In a recent study on young individuals with hyperglycemia, an inverse association between plasma glucose and GAL-1 levels was found [37]. This may be mediated by affecting monocyte function, possibly explaining the lower GAL-1 levels found in patients with T1D [26]. The relevance of GAL-1 in the development of CeD is poorly understood. Expression of GAL-1 in duodenal biopsies of patients with CeD is, thus far, decreased in untreated patients with CeD compared to control individuals [38]. Studies on the association between circulating GAL-1 levels and T cells in children with combined diagnosis of T1D and CeD are currently missing.

The aim of this project was, therefore, to study the association between immune markers and GAL-1 in clinical samples (sera) of children with T1D and/or CeD. Research questions included the following: (1) is there a positive correlation between GAL-1 and soluble immune markers, i.e. cytokines/chemokines, APP, and MMPs, in children with T1D and/or CeD; (2) is there a positive correlation between GAL-1 and T-cell subsets, specifically Th cells as well as Treg cells defined as CD4^+^CD25^hi^ also expressing CD39, CD45RA, CD101, and/or CD129, in children with T1D and/or CeD.

## Materials and methods

### Study cohort

#### Diagnostic criteria and examination procedures

This is a multi-center study that includes children with T1D and/or CeD or those with neither disease, collected at Linköping University Hospital, Linköping, Sweden and Ryhov County Hospital, Jönköping, Sweden, as previously described [39, 40].

The cohort of this study included a total of 103 children: 27 children with exclusively T1D, 18 children with a combined diagnosis of T1D and CeD, 16 children with exclusively CeD, and 42 children without these diseases, i.e. reference children. Study sample collection was conducted in two separate parts, Cohorts I and II (Table 1). Information regarding sex, age, and duration of disease (T1D and CeD) is summarized in Table 1.

**Table 1.** Characteristics of participants (whole group, sampling Cohort I and sampling Cohort II); children with T1D, CeD, T1D and CeD (T1D + CeD), and reference children (Control)

	*n*	Whole study cohort (I + II)	Cohort I	Cohort II
		103 (100%)	47 (46%)	56 (54%)
T1D	***n***	**27 (26%)**	**13 (28%)**	**14 (25%)**
	Sex, G (*n*)/B (*n)*	14/13	7/6	7/7
	Age (years)	13.0 (6.0–17.4)	11.0 (6.0–14.0)	14.8 (8.4–17.4)
	** **Girls	12.7 (8.4–17.2)	10.5 (10.0–14.0)	13.7 (8.4–17.2)
	Boys	13.5 (6.0–17.4)	12.0 (6.0–13.5)	16.8 (13.7–17.4)
	Duration T1D (years)	4.2 (0.3–12.9)	3.1 (0.3–10.9)	5.5 (1.6–12.9)
	Girls	5.7 (0.8–12.0)	3.1 (0.8–10.2)	6.0 (3.8–12.0)
	Boys	3.3 (0.3–12.9)	3.1 (0.3–10.9)	3.3 (1.6–12.9)
T1D + CeD	***n***	**18 (17%)**	**8 (14%)**	**10 (18%)**
	Sex, G (*n*)/B (*n*)	7/9 (2^a^)	3/5	4/4 (2^a^)
	Age (years)	11.5 (7.0–15.4)	11.3 (7.0–14.0)	9.6 (7.7–15.4)
	** **Girls	9.5 (7.5–13.2)	9.5 (7.5–12.5)	8.1 (7.7–13.2)
	Boys	12.0 (7.0–15.4)	12.0 (7.0–14.0)	11.9 (7.7–15.4)
	Duration T1D (years)	3.5 (0.1–10.8)	3.0 (1.2–7.9)	4.0 (0.1–10.8)
	Girls	2.6 (0.1–5.5)	2.4 (1.9–5.5)	3.6 (0.1–4.9)
	Boys	3.6 (1.2–10.8)	3.6 (1.2–7.9)	5.6 (3.0–10.8)
	Duration CeD (years)	2.1 (0.4–10.2)	0.9 (0.4–4.2)	3.6 (0.4–10.2)
	Girls	4.5 (0.5–10.2)	0.9 (0.5–1.9)	4.8 (4.5–10.2)
	Boys	1.9 (0.4–4.2)	0.8 (0.4–4.2)	2.1 (0.4–2.7)
CeD	***n***	**16 (15%)**	**10 (21%)**	**6 (11%)**
	Sex, G (*n*)/B (*n*)	10/6	6/4	4/2
	Age (years)	10.0 (7.4–17.3)	10.3 (8.0–14.5)	11.3 (7.4–17.3)
	** **Girls	11.5 (7.5–17.3)	11.5 (10.0–14.5)	14.5 (7.5–17.3)
	Boys	10.0 (7.4–10.5)	10.0 (8.0–10.5)	8.7 (7.4–9.9)
	Duration CeD (years)	5.9 (0.6–11.7)	6.5 (0.7–11.7)	3.7 (0.6–6.1)
	Girls	6.6 (0.7–11.7)	9.4 (0.7–11.7)	6.0 (1.4–6.1)
	Boys	1.0 (0.6–5.8)	2.6 (1.0–5.8)	0.7 (0.6–0.8)
Reference	***n***	**42 (40%)**	**16 (35%)**	**26 (46%)**
	Sex, G (*n*)/B (*n*)	19/19 (4^a^)	8/8	11/11 (4^a^)
	Age (years)	12.0 (6.8–16.8)	11.4 (8.0–14.5)	11.6 (6.8–16.8)
	** **Girls	12.0 (8.5–16.8)	12.0 (9.5–14.5) (1^a^)	10.0 (7.5–16.8)
	Boys	12.0 (6.8–16.8)	10.8 (8.0–12.0) (2^a^)	12.3 (6.8–16.4)

Age and/or sex data not available.

Age and diabetes duration are presented as median (minimum, maximum).

The cohort included a total of 103 children: 27 children with exclusively T1D (26% of the total cohort), 18 children with double diagnosis, i.e. T1D and CeD (17% of the total cohort), 16 children with exclusively CeD (15% of the total cohort) and 42 children without these diseases, i.e. reference children (40% of the total cohort). The collection of the study cohort was conducted in two separate parts. GAL-1 and soluble immune markers were analysed within both parts of the cohort (i.e. Cohorts I and II; 103 children), whereas Treg-associated markers were analysed only within the second part (i.e. Cohort II; 46 children). Information regarding sex, age, and duration of disease (T1D and CeD) are presented for the whole as well as Cohorts I and II, respectively.

Abbreviations: G: girls; B: boys.

T1D was diagnosed according to the International Society for Pediatric and Adolescent Diabetes (ISPAD) guidelines [41]: symptoms of diabetes plus casual plasma glucose concentration ≥ 11.1 mmol/L (200 mg/dL) or fasting plasma glucose ≥ 7.0 mmol/L (≥126 mg/dL) or 2-hour post-load glucose ≥ 11.1 mmol/L (≥200 mg/dL) during an oral glucose tolerance test). CeD was diagnosed according to the modified version of The European Society of Pediatric Gastroenterology and Nutrition (ESPGAN) criteria.

Duration of T1D was defined as the time from the date of diagnosis until the date of sample collection. The duration of CeD was the time from the date when biopsy confirmed diagnosis until the date of sample collection. All children in the double diagnosis group were diagnosed with T1D before diagnosis with CeD.

The control group consisted of healthy children (i.e. reference), and neither the reference children nor their first-degree relatives displayed any clinical signs of T1D, CeD, or other autoimmune diseases.

The general criteria for participating in the study was that the children in all study groups should not show any signs of allergy, colds, or other infections at the time of sample collection.

#### Sample collection for analysis of GAL-1, soluble immune markers, and Treg cells

GAL-1 and soluble immune markers were analysed in samples from both parts of the cohort (Cohort I + II, i.e. 103 children, Table 1). Sampling was conducted in connection with a routine visit to the pediatric clinic. One-hour prior to blood sampling, a local anesthetic ointment was placed on the arm. Blood samples (~5 ml) were collected in vacutainer tubes without anticoagulant (BD Biosciences, San Jose, CA). Thirty minutes after sampling, sera were separated by centrifugation of the whole blood samples at 2000 × *g* for 10 min. Thereafter sera were aliquoted and stored at −80°C until analysis. Multiple freeze-thaw cycles were avoided because this is detrimental to many soluble immune markers in sera.

Treg subsets were analysed in samples exclusively within Cohort II (Table 1). Staining for flow cytometry was performed in whole blood (~5 ml), supplemented with EDTA, within 24 hours of sampling, following the same procedure as described earlier [39].

#### Analysis of GAL-1

GAL-1 was analysed in sera of 103 children (according to Table 1) using a commercial human Galactin-1 enzyme-linked immunoassay (ELISA) kit with a sensitivity of 0.129 ng/ml according to the manufacturer’s instructions (Human Galectin-1 Immunoassay, Quantikine ELISA, R&D Systems, Minneapolis, MN). Intra- and inter-assay coefficient of variation were 7.1% and 9.5%, respectively. In brief, controls, standards, and diluted samples were added in duplicate into a 96-well microplate (100 μL per well) precoated with a polyclonal antibody specific for human GAL-1 and incubated for 2 hours at room temperature (RT). After aspiration and plate washing, horseradish-conjugated detection Human GAL-1 antibody was added (200 μL per well). The plate was incubated for another 2 hours at RT. Following this, the aspiration and wash step was repeated. Color reagents (substrate solution) were added (200 μL per well), and the plate was incubated for another 30 min at RT. Afterwards, the enzymatic reaction was stopped by adding a 50 μl stop solution (2 N H_2_SO_4_) per well. The optical density was measured on a plate reader set to 450 nm. The analysis was performed in duplicates.

Three internal controls (from R&D Systems) for GAL-1 were included in each assay as a control for intra- and inter-assay variation. The acceptance range for controls according to the manufacturer´s instructions was, for the low control: 0.91–2.66 ng/ml, for the medium control: 2.33–6.62 ng/ml and for the high control: 4.69–12.60 ng/ml.

#### Analysis of soluble immune markers

Twenty-eight immune markers, including cytokines and chemokines, adipocytokines, metalloproteinases, and APP, were analysed in sera from 103 children (Table 1) using multiplex fluorochrome sandwich immunoassays based on Luminex xMAP technology (Luminex, Bio-Rad Laboratories, Hercules, CA) Bio-Plex assays (Bio-Rad Laboratories) on the instrument Bio-Plex 200 system (Bio-Rad Laboratories) according to the manufacturer’s instructions [9]. The median fluorescence intensity (MFI) for each sample was registered and analysed with Bio-Plex Manager Software 5.0 (Bio-Rad Laboratories). The analyte concentrations were estimated using a five-parameter logistic model standard curve. Immune markers were sub-grouped and presented together with the cut-off values for minimum detectable concentrations in Supplementary 1.

Quality controls (recombinant protein supplied by the manufacturer) for each immune marker were included in each experiment to monitor the performance of the assay. All quality controls were within the expected range stated by the manufacturer. To evaluate the reproducibility of the assay, intra- and inter-assay coefficient of variation were calculated for the quality controls assayed in duplicate. The intra- and inter-assay variability in our analysis were 6.0% and 6.2%, respectively.

#### Analysis of Tregs and Treg subsets

Treg-associated markers were studied in a sub-sample cohort (i.e. study Cohort II, according to Table 1). For analysis of Tregs and Treg subsets, cell surface markers (CD4, CD25, CD39, CD45RA, CD101, CD129, and CD127) and intracellular FOXP3 cells were stained with fluorochrome-conjugated monoclonal antibodies. The analysis was performed on two panels with the following combinations of antibodies: (Panel 1) anti-CD39, anti-CD129, anti-CD45RA, anti-CD25, anti-CD101, and anti-CD4; (Panel 2) anti-CD39, anti-FOXP3, anti-CD45RA, anti-CD25, anti-CD127, and anti-CD4.

In samples exclusively stained for extracellular markers, the following was performed; blood samples were stained with antibodies for extracellular markers for 15 min at RT, thereafter, incubated for 10 min with 0.5 ml Optilyse C (Beckman Coulter, Manchester, UK), followed by 5 min incubation including 0.5 ml phosphate-buffered saline (PBS, Life Technologies, Stockholm, Sweden) and 0.5% bovine serum albumin (BSA, Life Technologies) and two final washes in PBS + 0.5% BSA.

For samples stained for both extra- and intracellular markers, the following was performed. Whole blood samples were stained with antibodies for extracellular markers for 15 min at RT and thereafter washed in PBS + 0.5% BSA. After discarding the cell supernatant, cells were resuspended and incubated with fixation/permeabilization buffer (eBioscience, San Diego, CA) for 30 min. Suspensions were thereafter centrifuged for 10 min at 500 × *g*, cell supernatants discarded, and cells washed with permeabilization buffer (eBioscience). Cells were resuspended in the permeabilization buffer and incubated for 30 min with antibodies for the intracellular marker. Cells were washed with PBS + 0.5% BSA, and cell supernatants were discarded. After staining, cells were resuspended with PBS + 0.5% BSA and kept at 4°C in darkness until analysis. Analysis was performed on a Gallios flow cytometer (Beckman Coulter, Manchester, UK).

#### Gating strategy and analysis

Lymphocytes were gated based on forward scatter and side scatter. From the lymphocyte gate, CD4^+^ cells were gated, followed by gating for CD25^+^ cells. From this population, a strict FOXP3^+^CD127^−^ gate was applied. This population was thereafter examined for the expression of CD39 and CD45RA. From the CD4^+^CD25^+^ gate, the 2% with the highest CD25 expression, CD4^+^CD25^hi^, was determined. The CD4^+^CD25^hi^ Treg population was then further examined for the expression of CD101 and CD129, as previously described [39].

Results were presented as the frequency of cells with expressed markers (%) or as MFI, equivalent to the amounts of extra- and intracellular markers on the cell. All analyses were coded and blinded, and subject-level analyses were not performed during processing in the software package Kaluza 1.2 (Beckman Coulter, Manchester, UK).

#### Analysis of autoantibodies

Autoantibodies directed against insulin (IA), glutamic acid decarboxylase (GAD_65_), tyrosine phosphatase (IA-2), zinc transporter 8 (ZnT8A) as biomarkers of T1D, and transglutaminase (tTGA) for CeD were analysed simultaneously using the highly sensitive Multiplex Antibody-Detection by Agglutination-PCR (ADAP) assay. In brief, sera were incubated with DNA-barcoded autoantigens whereas autoantibodies agglutinated to the autoantigens and formed a dense immune-complex. The dense immunocomplexes were further mixed with PCR amplification mixtures containing primers for all five autoantibodies and amplified for a total of 13 PCR cycles using On Deck Thermocycler (ODTC, Inheco, Martinsried, Germany). The amplified products were then mixed with cognate primer pairs for each autoantibody for specific quantification using real-time quantitative PCR (RT-qPCR). Roche LightCycler 480 System II (Roche Diagnostics International AG, Rotkreuz, Switzerland) was used to achieve a full sample-to-answer solution [42].

### Statistical analysis

All statistical analyses were conducted with GraphPad Prism 10.1 (GraphPad, San Diego, CA). Since the sample size was small and the results did not follow a Gaussian distribution, non-parametric tests were used. Spearman’s rank correlation test was used to compare paired observations in the different diagnosis groups. Z-transformation of individual samples was done by dividing the concentration of each marker with its standard deviation, summing the values included for each individual sample, and thereafter correlating the composite scores of immune markers with GAL-1 by Spearman’s correlation. Mann Whitney *U*-test was used to compare differences between two independent groups when the dependent variable was either ordinal or continuous but not normally distributed. Differences were considered statistically significant when *P* < 0.05.

### Ethics

This study was approved by the Research Ethics Committee of the Faculty of Health Sciences, Linköping University, Linköping, Sweden and the Regional Ethics Committee for Human Research, Linköping (approval number: Dnr 2006/M89-06 and complementary Dnr: 2012/27-32 and 2017/230-12) in accordance with the Helsinki Declaration.

Information adapted for their age was given both orally and written to all children, and their parents or responsible guardians. Prior to blood sampling, informed consent was received from all responsible guardians and adolescents.

## Results

### GAL-1 is positively correlated to age and height in children with T1D

In children diagnosed exclusively with T1D, GAL-1 was positively correlated to age (*r* = 0.38, *P* = 0.049, Supplementary 2a) as well as height (*r* = 0.63, *P* = 0.019, Supplementary 2b), a phenomenon not observed in any of the other studied groups within Cohort II. Contrary, GAL-1 was not correlated to sex, weight, BMI, or duration of T1D or HbA1c in children with T1D and/or CeD ( Supplementary 3). There was no correlation between the concentration of GAL-1 and concentrations of autoantibodies directed against GAD_65_, IA-2, insulin, ZnT8, and transglutaminase in children with exclusively T1D or CeD, a combination of T1D and CeD or without these diseases (reference children) (data not shown).

### GAL-1 is positively correlated to pro-inflammatory immune markers in children with combined T1D and CeD

**Figure 1.**
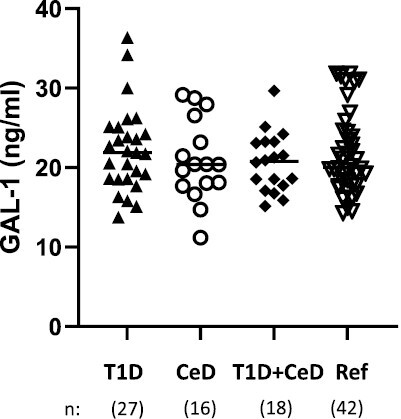
Concentration of GAL-1 did not differ between children diagnosed with T1D and/or CeD, compared to reference (Ref) children.

The serum level of GAL-1 did not differ between the groups of children with either T1D and/or CeD nor compared to reference children (Fig. 1). However, the concentration of GAL-1 was correlated to several soluble immune markers relevant for both T1D and CeD, as summarized in Table 2.

**Table 2.** Correlations between GAL-1 and other soluble immune markers

Subgroup	Immune marker	T1D *n* = 27		T1D and CeD *n* = 18		CeD *n* = 16		Reference *n* = 42	
		*r*-value	*P*-value	*r*-value	*P*-value	*r*-value	*P*-value	*r*-value	*P*-value
Th1	IFN-γ			*0.40*	*0.097*				
Th17	IL-22			*0.46*	*0.058*				
	IL-33					*0.44*	*0.090*		
Treg	IL-10			*0.42*	*0.086*				
Growth factor	G-CSF			*0.40*	*0.100*				
Pro-inflammatory	IL-1β	**0.43**	**0.027**	**0.56**	**0.016**				
	IL-6			**0.54**	**0.021**				
	IL-8	**0.43**	**0.026**					**0.36**	**0.020**
	TNF-α			**0.69**	**0.002**				
Adipocytokine	Visfatin			*0.42*	*0.081*				
MMPs	MMP-1			*0.41*	*0.090*	*0.44*	*0.090*		
	MMP-2							**0.41**	**0.007**
	MMP-3			*0.41*	*0.100*	**0.53**	**0.039**		

GAL-1 is positively correlated with several of the immune markers associated with T helper (Th) 1 (IFN-γ) and Th17 (IL-22 and IL-33), Treg (IL-10), growth factor (G-CSF), pro-inflammatory (IL-1β, IL-6, IL-8, TNF-α), adipocytokine (visfatin), and matrix metalloproteinases (MMP-1, MMP-2, and MMP-3) in children exclusively with type 1 diabetes (T1D) and/or celiac disease (CeD), compared to the reference children.

*P*-values <.05 are marked in bold and *P*-values ≥.05 are marked in italic.

**Figure 2.**
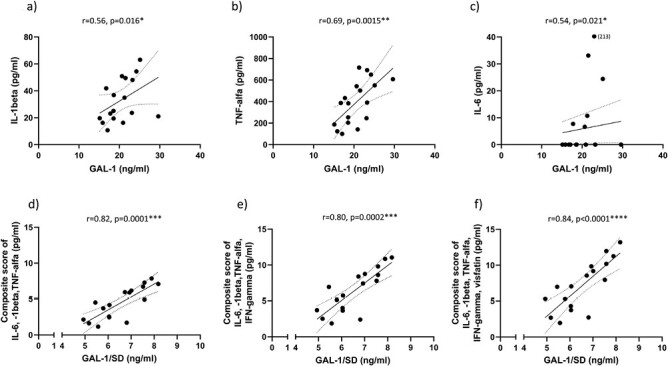
Concentration of GAL-1 in children diagnosed with both T1D and CeD were positively correlated to secretion of the pro-inflammatory biomarkers IL-1β (*r* = 0.56, *P* = 0.016, **a**), TNF-α (*r* = 0.69, *P* = 0.0015, **b**), and IL-6 (*r* = 0.54, *P* = 0.021, **c**) (whole study cohort, *n* = 18). Composite scores combining z-transformed values of IL-1β, IL-6, and TNF-α (*r* = 0.82, *P* = 0.0001, **d**); IL-1β, IL-6, TNF-α, and IFN-γ (*r* = 0.80, *P* = 0.0002, **e**); and IL-1β, IL-6, TNF-α, IFN-γ, and visfatin (*r* = 0.84, *P* < 0.0001, **f**) were all correlated to GAL-1 (divided with its standard deviation) in children with combined T1D and CeD (*n* = 17, one outlier excluded due to a high value of GAL-1). 95% confidence intervals are indicated in all figures.

The concentration of GAL-1 in children diagnosed with both T1D and CeD was positively correlated to the concentration of the pro-inflammatory immune markers IL-1β (*r* = 0.56, *P* = 0.016, Fig. 2a), TNF-α (*r* = 0.69, *P* = 0.0015, Fig. 2b), and IL-6 (*r* = 0.54, *P* = 0.021, Fig. 2c). Clustering of IL-1β, IL-6, and TNF-α by Z-transformation indicated a strong positive correlation with GAL-1 (*r* = 0.82, *P* = 0.0001, Fig. 2d).

Even though not significant, Th1-associated IFN-γ and T1D-associated visfatin tended to be positively correlated to GAL-1 in children with combined T1D and CeD (Table 2). Clustering of IL-1β, IL-6, TNF-α, and IFN-γ was positively correlated with GAL-1 (*r* = 0.80, *P* = 0.0002, Fig. 2e) and furthermore, the cluster of IL-1β, IL-6, TNF-α, IFN-γ, and visfatin had a strong, positive correlation with GAL-1 (*r* = 0.84, *P* < 0.0001, Fig. 2f).

Several other immune markers, including the growth factor G-CSF, Treg-associated (IL-10), Th17-associated (IL-22), and MMPs (MMP-1 and MMP-3) tended to be positively correlated to GAL-1 in children with combined T1D and CeD (Table 2).

In children diagnosed with exclusively T1D, GAL-1 was positively correlated to IL-β (*r* = 0.54, *P* = 0.027, Supplementary 4a). GAL-1 was positively correlated to IL-8 in both T1D (*r* = 0.43, *P* = 0.026, Supplementary 4b) and reference children (*r* = 0.36, *P* = 0.02, Table 2). Only MMPs were positively correlated to GAL-1 in children with CeD (MMP-3: *r* = 0.53, *P* = 0.039) or reference children (MMP-2: *r* = 0.41, *P* = 0.007), respectively ( Supplementary Fig. 4c and d, respectively).

All data for the soluble immune markers correlating to one or more groups are presented in Supplementary 5a. The remaining studied immune markers (IL-5, IL-9, IL-13, IL-15, IL-17A, IL-25, monocyte chemoattractant protein-1, MIP-1α, MIP-1β, PCT, Ferritin, tPA, fibrinogen, SAA, and resistin) were not correlated to GAL-1 in any of the four studied groups.

### GAL-1 is positively correlated to Tregs and Treg subsets exclusively in children with T1D

**Figure 3.**
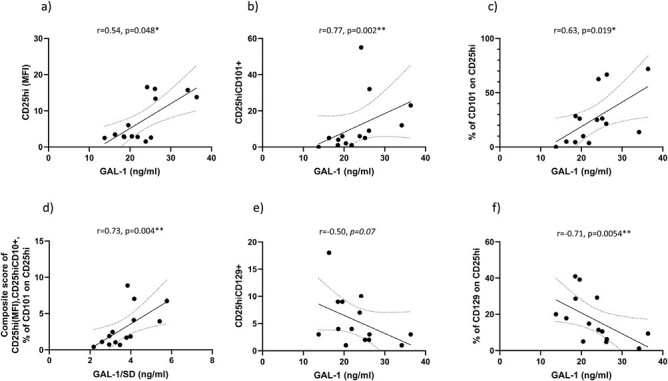
Population of CD25^hi^ T cells (*r* = 0.54, *P* = 0.048, **a**), population of CD25^hi^CD101^+^ (*r* = 0.77, *P* = 0.002, **b**), and ratio of CD101^+^ cells among CD25^hi^ T-cell population (*r* = 0.63, *P* = 0.019, **c**) were positively correlated to concentration of GAL-1 in children with T1D (*n* = 14, Cohort II). A composite score of CD25^hi^, the population of CD25^hi^CD101^+^, and the frequency of CD101 positive cells among the CD25^hi^ T-cell population was positively correlated with GAL-1 (divided with its standard deviation; *r* = 0.73, *P* = 0.004, **d**). Contrary, the population of CD25^hi^CD129^+^ (*r* = −0.50, *P* = 0.07, **e**) and the frequency of CD129 positive cells among CD25^hi^ T cells (*r* = −0.71, *P* = 0.0054, **f**) were both inversely correlated to the concentration of GAL-1 in children with exclusively T1D. 95% confidence intervals are indicated in all figures.

GAL-1 was positively correlated to Treg cells, exclusively in T1D children, as summarized in Table 3. In children diagnosed with only T1D, the population of CD25^hi^ Treg cells was positively correlated to the concentration of GAL-1 (*r* = 0.54, *P* = 0.048, Fig. 3a). Furthermore, in T1D children, the population of CD25^hi^CD101^+^, as well as the frequency of CD101 expressing CD25^hi^ Treg cells, were both positively correlated to the concentration of GAL-1 [*r* = 0.77, *P* = 0.002 (Fig. 3b) and *r* = 0.63, *P* = 0.019 (Fig. 3c), respectively]. Clustering of CD25^hi^ (MFI), the population of CD25^hi^CD101^+^ and % of CD101 expressing cells among the CD25^hi^ T-cell population was positively correlated with GAL-1 (*r* = 0.73, *P* = 0.004, Fig. 3d) in T1D children.

**Table 3.** Correlations between GAL-1 and subsets of Treg cells

Subgroup	Immune marker	T1D *n* = 14		T1D and CeD *n* = 8		CeD *n* = 6		Reference *n* = 8	
		*r*-value	*P*-value	*r*-value	*P*-value	*r*-value	*P*-value	*r*-value	*P*-value
CD25/CD127/FOXP3	% CD4^+^CD25^hi^			*0.71*	*0.059*				
	MFI of CD25 on CD25^hi^	**0.54**	**0.048**	*0.63*	*0.099*				
	MFI of FOXP3 on CD4^+^CD25^+^FOXP3^+^CD127-					**0.94**	**0.017**		
	MFI of FOXP3 on CD4^+^CD25^+^CD127^−^CD45RA^+^					*0.77*	*0.103*	*0.64*	*0.096*
CD25/CD101	CD25^hi^CD101^+^	**0.77**	**0.002**						
	% CD101 of CD25^hi^	**0.63**	**0.019**	*−0.72*	*0.052*				
CD25/CD129	CD25^hi^CD129^+^	*−0.50*	*0.070*						
	% CD129 of CD25^hi^	**−0.71**	**0.005**						

GAL-1 is correlated with several subsets of Treg cells (CD4^+^, CD25^+^, CD101^+^, CD127^−^, CD129^+^, and FOXP3^+^) in children exclusively with T1D and/or CeD, compared to the reference children.

*P*-values <.05 are marked in bold and *P*-values ≥.05 are marked in italic.

Abbreviation: MFI, median fluorescence intensity.

Contrary, the population of CD25^hi^CD129^+^ and the frequency of CD129 positive cells among CD25^hi^ T cells were both inversely correlated to the concentration of GAL-1 (*r* = -0.50, *P* = 0.07 and *r* = -0.71, *P* = 0.0054, Fig. 3e and f, respectively) only in children with T1D. The same pattern of a relation between the concentration of GAL-1 and Treg cells was not observed within the other studied groups ( Supplementary 5b).

## Discussion

We examined the association between circulating GAL-1 and several soluble immune markers (cytokines, APPs, adipocytokines, and MMPs) measured in blood sera and markers of Treg cells measured through flow cytometry in children with T1D and/or CeD and children without these diagnoses. While there were no differences in serum levels of GAL-1 between the groups, we found that GAL-1 presents distinctly different correlations with soluble immune markers and Treg cells, between children diagnosed with T1D and/or CeD. Most striking is that children diagnosed exclusively with T1D had correlation patterns distinct from that observed in children diagnosed with both T1D and CeD.

Studies of circulating GAL-1 levels in human T1D are currently scarce, and we are not aware of any other study detecting GAL-1 specifically in children with T1D or CeD. Here, we find that high GAL-1 correlates positively with elevated IL-1β and IL-8 in children with exclusively T1D. It has previously been reported that IL-8 is higher in adolescents with T1D and poor metabolic control, possibly providing explanation to this observation [43]. Furthermore, IL-1β and IL-8 were recently reported to be elevated together in the vitreous fluid of patients with diabetes and proliferative retinopathy [44]. Several studies report a role for GAL-1 in diabetic retinopathy, and it is possible that our observation is indicative of a shared regulation for IL-1β and IL-8 and GAL-1 in the microvascular pathophysiology of T1D [34, 45, 46]. In addition, an intervention study administering an IL-1β receptor antagonist to a small sample of patients with T1D indicated anti-inflammatory effects on mononuclear cells through an IL-8–mediated pathway [47]. Thus, specific correlations between GAL-1, IL-1β, and IL-8 could suggest a disease-specific pathway in which GAL-1 interacts in T1D.

In children with a combined diagnosis of T1D and CeD, GAL-1 correlated positively with IL-1β, IL-6, and TNF-α. The correlation between GAL-1 and IL-1β is consistent with our observations in children with only T1D, providing further support to a more disease-specific association in T1D. Furthermore, circulating GAL-1 has previously been shown to correlate positively with IL-6 and TNF-α in a community-based study sample of 989 adult participants, and it is possible that these correlations are induced by ongoing metabolic inflammation or less disease-specific inflammatory processes [48]. We are not aware of any previous reports on a positive correlation between high GAL-1 and elevated IL-6 and TNF-α in children. However, our observations indicate that GAL-1 may interact with these cytokines in children, as previously reported in adults. Analyses in children with a double diagnosis also demonstrated a strong positive correlation between GAL-1 and composite estimates of several cytokines, with the strongest positive correlation found when combining IL-1β, IL-6, and TNF-α, but also a positive correlation to Th1-associated IFN-γ and the T1D-associated immune marker visfatin. T1D and CeD share several common risk factors, including common risk-associated human leukocyte antigen (HLA)-haplotypes, and microbiotic alterations in the gut [49]. However, there are both common and unique risk factors for T1D and CeD, and it may be that children with a double diagnose present certain HLA-haplotypes and lack of gut microbiota diversity to a larger degree than those with only one diagnosis. This may explain the specific associations found between GAL-1 and other immune markers.

In children with CeD, GAL-1 correlated positively with MMP-3 but not with any other immune marker evaluated in this study. MMP-3 has previously been associated with CeD, and with T cell–mediated mucosal destruction in CeD [50, 51]. Indeed, GAL-1 is increased in cellular processes of tissue remodeling, and it is possible that correlation with MMP-3 is associated with other intestinal cells, such as myofibroblasts [52, 53]. The absence of correlations found in children with a double diagnosis suggests that it is not CeD, but the joint auto-immune burden, which explains the correlations between GAL-1 and immune markers in children with both T1D and CeD.

The functional role of GAL-1 as a regulator of Treg cells is well-described [24, 54], and Treg cells are reported to have a preservative role on β cell function in T1D [55]. Here, GAL-1 did not correlate with the Treg markers CD4 or CD25 in children with T1D or CeD. However, in children with T1D, GAL-1 correlated positively with CD25 MFI in Treg cells, as well as with both the number and frequency of CD101^+^ Treg cells. CD101 has previously been identified as a T1D susceptibility gene and is proposed to restrain the expansion of diabetogenic CD4 and CD8 expressing T lymphocytes and reduce T1D frequency in NOD mice [56]. It is thus possible that interaction between GAL-1 and CD101 is a shared pathway in the development of T1D. Studies exploring any direct interaction between GAL-1 and CD101 should therefore be conducted.

CD129 is also suggested to play an important role in the development of intrathymic T cells [57]. We found an inverse correlation between the frequency of CD129^+^ Treg cells and GAL-1 in children with exclusively T1D. The implication of the inverse correlation between GAL-1 and CD129 is not evident. It may be that the correlation reflects the previously well-known association between GAL-1 and Tregs [24, 31]. However, we cannot explain, without further mechanistic studies, how GAL-1 may interact with CD129 in children with T1D.

In children with combined T1D and CeD, the positive correlation between GAL-1 and MFI of CD25 in Tregs presented a higher *r*-value and a *P*-value of 0.099, which is similar to the statistically significant correlation seen in children with only T1D. It is possible that this correlation was not significant in children with both diseases because of a smaller sample size. It is also possible that GAL-1 varies with anthropometric variables secondary to other underlying mechanisms such as puberty, sex, or comorbidities. Future studies on children with both T1D and CeD, with larger sample sizes, may provide insight of whether GAL-1 correlates with percentage of CD101 in CD25^hi^ in children with double diagnosis.

In children with CeD, GAL-1 was positively correlated with MFI of FOXP3 in FOXP3^+^CD127^−^ Tregs, although in a very small sample size. Analysis of FOXP3 expression in combination with CD39 is proposed as a marker of dysfunctional Treg cells in CeD [18], while another study indicates that FOXP3 may be associated specifically in adult patients with refractory CeD [58]. Collectively, circulating GAL-1 was associated with markers of Tregs in both T1D and CeD.

In children with a combined diagnosis of T1D and CeD, GAL-1 correlated positively with both age and height. A previous study on children with and without obesity did not find any correlation between GAL-1 and age [37]. The same study reported a correlation between GAL-1 and both waist circumference and fat mass, specifically in children with obesity. It is possible that GAL-1 varies with anthropometric variables secondary to other underlying mechanisms such as puberty status, sex, or comorbidities.

Regarding limitations, this study only recruited patients from Swedish hospitals and studies of patients from other parts of the world may have other findings. Nonetheless, our study did recruit both girls and boys with a diverse age range. The cross-sectional design of the study prevented any causal conclusions regarding observed correlations. While we found several interesting correlations between GAL-1 and different immunity markers in the different study groups, several of these markers are not specific to a given cell type. Thus, the action of GAL-1 may involve different cell types and different signaling pathways within each study group. The sample sizes for studies on Tregs vary between groups of children, and for some groups the n-value is small. This may lead to type 2 errors, where some observations may be missed due to a lack of statistical power. Even so, we can report interesting correlations that link GAL-1 to immunity markers and Treg cells, specifically in T1D and CeD, and therefore added an additional piece to the puzzle of dysregulated immunity in T1D and CeD. Our observations should now be validated in future studies, including larger samples of any given diagnosis. Future studies should also explore the direct interactions between GAL-1 and Treg cells, specifically in the context of T1D.

In summary, although serum levels of GAL-1 did not differ between the groups, we found distinct differences between GAL-1 and correlations with soluble immune markers and Treg cells, between children diagnosed with T1D and/or CeD. Children diagnosed with both T1D and CeD showed a pattern of strong positive correlation between GAL-1 and several cytokines associated with inflammation, possibly explained by a more pronounced auto-immune burden. The specific correlations with IL-1β and IL-8 in T1D could suggest a more pathway-specific role for GAL-1 in T1D. Furthermore, children diagnosed with exclusively T1D showed a different pattern with positive correlations between GAL-1 and Treg-associated markers, possibly indicating that GAL-1 interacts with Treg cells in human T1D. Thus, GAL-1 might have a modulating role in inflammatory activity seen in children with exclusively T1D. The relation between GAL-1 and the immune markers studied was limited in children with only CeD and may be of less significance than the observations found in T1D. Going forward, a possible role for GAL-1 in IL-1β and IL-8 signaling should be further examined by mechanistic studies in T1D to determine a potential mediating role. Furthermore, the influence of HLA-haplotype and microbiota composition on GAL-1 levels in children with multiple autoimmune diseases should also be further studied, as this could provide new insights into how these factors convey risk. This far, we can conclude that the association observed between GAL-1, soluble immune markers, and Treg cells may indicate a role for GAL-1 in the pathophysiology of T1D and, to some extent, also in CeD.

## Supplementary Material

uxad131_suppl_Supplementary_Materials

## Data Availability

The data underlying this article will be shared upon reasonable request to the corresponding author.

## Abbreviations

APP: acute-phase proteins
CD: cluster of differentiation
CeD: celiac disease
GAL-1: galectin-1
IFN: interferon
IL: interleukin
MFI: median fluorescence intensity
MIP: macrophage inflammatory proteins
MMP: matrix metalloproteinases
NOD: nonobese diabetic
SAA: serum amyloid A
T1D: type 1 diabetes
Th: T helper
TNF: tumor necrosis factor
Treg: T regulatory
tPA: tissue protein activator
