# Supplementary material for: Galectin-1 correlates with inflammatory markers and T regulatory cells in children with type 1 diabetes and/or celiac disease
Source: Clin Exp Immunol. 2023 Dec 13;215(3):240–50. doi: 10.1093/cei/uxad131 (PMC10876110; doi:10.1093/cei/uxad131)
Supplement: uxad131_suppl_Supplementary_Materials [file uxad131_suppl_supplementary_materials.zip › uxad131_suppl_Supplementary_Data_S5.docx]

**Supplement 5a** Correlations between Galectin-1 and soluble immune markers

| **Immune marker** |  | **T1D** | | |  | **T1D and Celiac disease** | | | |  | **Celiac disease** | | | | | | |  | **Reference** | | | | | |
| --- | --- | --- | --- | --- | --- | --- | --- | --- | --- | --- | --- | --- | --- | --- | --- | --- | --- | --- | --- | --- | --- | --- | --- | --- |
|  |  | ***r*** | ***p*** | *n* |  | ***r*** | ***p*** | *n* | |  | ***r*** | | ***p*** | | *n* | | |  | ***r*** | | ***p*** | | *n* | |
| ***Th1 associated*** |  | |  | |  | |  | |  | | |  | | | |  | | | |  | | | |  |
| IFN-γ |  | 0.23 | 0.25 | 27 |  | ***0.40*** | ***0.097*** | 18 |  | - 0.078 | | 0.77 | | 16 | |  | 0.094 | | | 0.55 | | 42 | |  |
| **Treg *associated*** |  | |  | |  | |  | |  | | |  | | | |  | | | |  | | | |  |
| IL-10 |  | 0.31 | 0.12 | 27 |  | *0.42* | *0.086* | 18 |  | 0.21 | | 0.41 | | 16 | |  | 0.17 | | | 0.27 | | 42 | |  |
| **Th17 *associated*** |  | |  | |  | |  | |  | | |  | | | |  | | | |  | |  | |  |
| IL-22 |  | -0.07 | 0.73 | 27 |  | ***0.46*** | ***0.058*** | 18 |  | 0.05 | | 0.86 | | 16 | |  | -0.02 | | | 0.88 | | 42 | |  |
| IL-33 |  | 0.07 | 0.73 | 27 |  | *0.07* | *0.79* | 18 |  | *0.44* | | *0.093* | | *16* | |  | 0.24 | | | 0.13 | | 42 | |  |
| **Growth factor** |  | |  | |  | |  | |  | | |  | | | |  | | | |  | | | |  |
| G-CSF |  | 0.04 | 0.83 | 27 |  | *0.40* | *0.10* | 18 |  | 0.24 | | 0.37 | | 16 | |  | - 0.068 | | | 0.67 | | 42 | |  |
| **Pro-inflammatory** |  |  |  |  |  |  |  |  |  |  | |  | |  | |  |  | | |  | |  | |  |
| IL-1β |  | **0.43** | **0.027** | 27 |  | **0.56** | **0.016** | 18 |  | 0.15 | | 0.58 | | 16 | |  | 0.18 | | | 0.26 | | 42 | |  |
| IL-6 |  | 0.25 | 0.22 | 27 |  | **0.54** | **0.021** | 18 |  | 0.34 | | 0.20 | | 16 | |  | - 0.036 | | | 0.82 | | 42 | |  |
| IL-8 |  | **0.43** | **0.026** | 27 |  | 0.33 | 0.17 | 18 |  | - 0.20 | | 0.46 | | 16 | |  | **0.36** | | | **0.020** | | 42 | |  |
| TNF-α |  | 0.10 | 0.62 | 27 |  | **0.69** | **0.002** | 18 |  | 0.19 | | 0.48 | | 16 | |  | 0.15 | | | 0.33 | | 42 | |  |
| **Adipocytokines** |  | |  | |  | |  | |  | | |  | | | |  | | | |  | | | |  |
| Visfatin |  | 0.32 | 0.11 | 27 |  | *0.42* | *0.081* | 18 |  | 0.19 | | 0.48 | | 16 | |  | 0.21 | | | 0.17 | | 42 | |  |
| **MMPs** |  | |  | |  | |  | |  | | |  | | | |  | | | |  | | | |  |
| MMP-1 |  | 0.12 | 0.55 | 27 |  | *0.41* | *0.09* | 18 |  | *0.44* | | *0.091* | | *16* | |  | 0.049 | | | 0.76 | | 42 | |  |
| MMP-2 |  | 0.12 | 0.55 | 27 |  | *0.28* | *0.27* | 18 |  | - 0.16 | | 0.56 | | 16 | |  | **0.41** | | | **0.007** | | 42 | |  |
| MMP-3 |  | 0.13 | 0.52 | 27 |  | *0.41* | *0.10* | 18 |  | **0.53** | | **0.039** | | 16 | |  | 0.11 | | | 0.50 | | 42 | |  |

**Supplement 5b** Correlations between Galectin-1 and subsets of T-regulatory cells

| **Immune marker** |  | **T1D** | | | | | | | |  | | **T1D and Celiac disease** | | | | | |  | **Celiac disease** | | | | |  | | **Reference** | | |
| --- | --- | --- | --- | --- | --- | --- | --- | --- | --- | --- | --- | --- | --- | --- | --- | --- | --- | --- | --- | --- | --- | --- | --- | --- | --- | --- | --- | --- |
|  |  | ***r*** | | ***p*** | | | *n* | | |  | | ***r*** | | | ***p*** | *n* | |  | ***r*** | ***p*** | *n* | | |  | | ***r*** | ***p*** | *n* |
| **Tregs and Treg subsets** |  |  | |  | | |  | | |  | |  | | |  |  | |  |  |  |  | | |  | |  |  |  |
| ***CD25/CD127/FOXP3*** |  |  | |  | | |  | | |  | |  | | |  |  | |  |  |  |  | | |  | |  |  |  |
| CD24^+^CD25^hi^ |  | 0.43 | | 0.12 | | | 14 | | |  | | *0.71* | | | *0.059* | 8 | |  | - 0.29 | 0.58 | 6 | | |  | | 0.20 | 0.44 | 18 |
| MFI CD25^hi^ |  | **0.54** | | **0.048** | | | 14 | | |  | | *0.63* | | | *0.099* | 8 | |  | 0.029 | 0.99 | 6 | | |  | | - 0.046 | 0.85 | 18 |
| MFI FoxP3 of CD4^+^CD25^+^FoxP3^+^CD127^-^ |  | 0.20 | | 0.60 | | | 9 | | |  | | *0.58* | | | *0.24* | 6 | |  | **0.94** | **0.017** | 6 | | |  | | - 0.14 | 0.75 | 8 |
| % CD39^+^CD45RA^+^ of CD4^+^CD25^+^FoxP3^+^CD127^-^ |  | - 0.12 | | 0.78 | | | 9 | | |  | | *- 0.11* | | | *0.82* | 7 | |  | 0.71 | 0.14 | 6 | | |  | | 0.48 | 0.24 | 8 |
| MFI FOXP3 of CD4^+^CD25^+^CD127^-^CD45RA^+^ |  | 0.23 | | 0.55 | | | 9 | | |  | | *- 0.70* | | | *0.23* | 5 | |  | 0.77 | 0.10 | 6 | | |  | | 0.64 | 0.096 | 8 |
| ***CD25/CD101*** |  | | |  | |  | | |  | | |  | |  | | |  |  |  |  | |  |  | |  | |  |  |
| CD25^hi^CD101^+^ |  | **0.77** | **0.002** | | 14 | | |  | | | - 0.42 | | 0.93 | | | 8 | |  | 0.32 | 0.56 | | 6 |  | | 0.092 | | 0.72 | 18 |
| MFI CD101 of CD25^hi^ |  | 0.17 | 0.56 | | 14 | | |  | | | - 0.036 | | 0.95 | | | 8 | |  | - 0.26 | 0.71 | | 6 |  | | 0.29 | | 0.25 | 18 |
| % CD101 of CD25^hi^ |  | **0.63** | **0.019** | | 14 | | |  | | | *- 0.72* | | *0.052* | | | 8 | |  | 0.71 | 0.14 | | 6 |  | | 0.040 | | 0.87 | 18 |
| ***CD25/CD129*** |  | | |  | |  | | |  | | |  | |  | | |  |  |  |  | |  |  | |  | |  |  |
| CD25^hi^CD129^+^ |  | *-0.50* | *0.070* | | 14 | | |  | | | *- 0.17* | | 0.69 | | | 8 | |  | - 0.029 | 0.99 | | 6 |  | | 0.061 | | 0.81 | 18 |
| MFI CD129 of CD25^hi^ |  | 0.47 | 0.090 | | 14 | | |  | | | - 0.47 | | 0.25 | | | 8 | |  | - 0.14 | .80 | | 6 |  | | - 0.20 | | 0.42 | 18 |
| % CD129 of CD25^hi^ |  | **- 0.71** | **0.0054** | | 14 | | |  | | | - 0.40 | | 0.33 | | | 8 | |  | 0.086 | 0.92 | | 6 |  | | 0.022 | | 0.93 | 18 |

MFI=median fluorescence intensity
