# Supplementary material for: Galectin-1 correlates with inflammatory markers and T regulatory cells in children with type 1 diabetes and/or celiac disease
Source: Clin Exp Immunol. 2023 Dec 13;215(3):240–50. doi: 10.1093/cei/uxad131 (PMC10876110; doi:10.1093/cei/uxad131)
Supplement: uxad131_suppl_Supplementary_Materials [file uxad131_suppl_supplementary_materials.zip › uxad131_suppl_Supplementary_Material.docx]

**Legend to Supplements**

**Supplement 1**

Soluble immune markers are subdivided into the following categories: cytokines and chemokines (T helper (Th) -1, -2, -17, T regulatory (Treg), growth factor, pro-inflammatory, acute phase proteins (APPs), adipocytokines and matrix metalloproteinases (MMPs) and cut-off values for minimum detectable concentration are presented for each immune marker in pg/ml.

**Supplement 2**

Galectin-1 (GAL-1) was positively correlated to age (r=0.38, p=0.049, Suppl 2a) and height (r=0.63, p=0.019, Suppl 2b) in children diagnosed with exclusively type 1 diabetes (T1D).

95% confidence intervals are indicated in all figures.

**Supplement 3**

Correlations between GAL-1 and demographics, i.e., age, sex, height, weight, body mass index (BMI), duration of type 1 diabetes (T1D) and HbA1c in cohort II.

n.d. = not detected

**Supplement 4**

GAL-1 was positively correlated to IL-β (r=0.43, p=0.027, Suppl 3a) and IL-8 (r=0.43, p=0.026, Suppl 3b) in children diagnosed with exclusively type 1 diabetes (T1D). Only matrix metalloproteinases were positively correlated to galectin-1 in children with celiac disease (CeD) (MMP-3: r=0.53, p=0.39, Suppl 3c) or reference children (MMP-2: r=0.41, p=0.007, Suppl 3d) within the whole cohort.

95% confidence intervals are indicated in all figures.

**Supplement 5**

All data for the immune markers that were correlated to one or more groups within the study cohort are presented accordingly.

1. Correlations between GAL-1 and soluble immune markers
2. Correlations between GAL-1 and subgroups of T-regulatory cells
