# Supplementary material for: Galectin-1 correlates with inflammatory markers and T regulatory cells in children with type 1 diabetes and/or celiac disease
Source: Clin Exp Immunol. 2023 Dec 13;215(3):240–50. doi: 10.1093/cei/uxad131 (PMC10876110; doi:10.1093/cei/uxad131)
Supplement: uxad131_suppl_Supplementary_Materials [file uxad131_suppl_supplementary_materials.zip › uxad131_suppl_Supplementary_Data_S1.docx]

**Supplement 1.** Cut-off values for the analyzed soluble immune markers.

| Soluble immune markers | Cut-off |
| --- | --- |
|  |  |
| Cytokines and chemokines |  |
| Th1 associated |  |
| IFN-γ | 19.30 pg/mL |
| Th2 associated |  |
| IL-5 | 0.80 pg/mL |
| IL-9 | 0.70 pg/mL |
| IL-13 | 2.10 pg/mL |
| Th17 associated |  |
| IL-17A | 0.49 pg/mL |
| IL-22 | 0.30 pg/mL |
| IL-25 | 0.07 pg/mL |
| IL-33 | 0.58 pg/mL |
| Treg associated |  |
| IL-10 | 0.90 pg/mL |
| Growth factor |  |
| G-CSF | 1.10 pg/mL |
| Pro-inflammatory |  |
| IL-1β | 0.80 pg/mL |
| IL-6 | 1.10 pg/mL |
| IL-8 | 0.50 pg/mL |
| IL-15 | 4.20 pg/mL |
| MCP-1 | 6.70 pg/mL |
| MIP-1α | 2.40 pg/mL |
| MIP-1β | 1.10 pg/mL |
| TNF-α | 3.00 pg/mL |
|  |  |
| Acute phase proteins (APPs) |  |
| Ferritin | 1.30 ng/mL |
| Fibrinogen | 2.80 ng/mL |
| Procalcitonin (PCT) | 11.30 ng/mL |
| Serum Amyloid A (SAA) | 1.10 pg/mL |
| tissue Protein activator (tPA) | 6.15 ng/mL |
|  |  |
| Adipocytokines |  |
| Resistin | 1.00 pg/mL |
| Visfatin | 8.00 pg/mL |
|  |  |
| Matrix Metalloproteinases (MMPs) |  |
| MMP-1 | 33.70 pg/mL |
| MMP-2 | 39.70 pg/mL |
| MMP-3 | 28.50 pg/mL |
