# Supplementary material for: Galectin-1 correlates with inflammatory markers and T regulatory cells in children with type 1 diabetes and/or celiac disease
Source: Clin Exp Immunol. 2023 Dec 13;215(3):240–50. doi: 10.1093/cei/uxad131 (PMC10876110; doi:10.1093/cei/uxad131)
Supplement: uxad131_suppl_Supplementary_Materials [file uxad131_suppl_supplementary_materials.zip › uxad131_suppl_Supplementary_Data_S3.docx]

**Supplement 3** Correlations between Galectin-1 and demographics in cohort II

| **Demographics** | **Type 1 diabetes** | | | | | **Type 1 diabetes and**  **celiac disease** | | | | **Celiac disease** | | | | **Control** | | |
| --- | --- | --- | --- | --- | --- | --- | --- | --- | --- | --- | --- | --- | --- | --- | --- | --- |
|  |  | ***r*** | ***p*** | ***n*** |  | ***r*** | ***p*** | ***n*** |  | ***r*** | ***p*** | ***n*** |  | ***r*** | ***p*** | ***n*** |
| Age |  | **0.38** | **0.049** | 27 |  | 0.24 | 0.37 | 16 |  | 0.34 | 0.20 | 16 |  | - 0.01 | 0.94 | 35 |
| Sex |  | - | 0.43 | 27 |  | - | 0.94 | 16 |  | - | 0.26 | 16 |  | - | 0.53 | 35 |
| Height |  | **0.63** | **0.019** | 14 |  | 0.23 | 0.59 | 8 |  | n.d. | n.d. | n.d. |  | n.d. | n.d. | n.d. |
| Weight |  | 0.08 | 0.80 | 14 |  | 0.43 | 0.29 | 8 |  | n.d. | n.d. | n.d. |  | n.d. | n.d. | n.d. |
| BMI |  | -0.18 | 0.52 | 14 |  | 0.41 | 0.31 | 8 |  | n.d. | n.d. | n.d. |  | n.d. | n.d. | n.d. |
| Duration (T1D) |  | 0.15 | 0.47 | 27 |  | -0.09 | 0.75 | 8 |  | n.d. | n.d. | n.d. |  | n.d. | n.d. | n.d. |
| HbA_1_c |  | 0.34 | 0.23 | 14 |  | -0.09 | 0.83 | 8 |  | n.d. | n.d. | n.d. |  | n.d. | n.d. | n.d. |
