# Supplementary figures and images for: Galectin-1 correlates with inflammatory markers and T regulatory cells in children with type 1 diabetes and/or celiac disease
Source: Clin Exp Immunol. 2023 Dec 13;215(3):240–50. doi: 10.1093/cei/uxad131 (PMC10876110; doi:10.1093/cei/uxad131)

## Slide 1
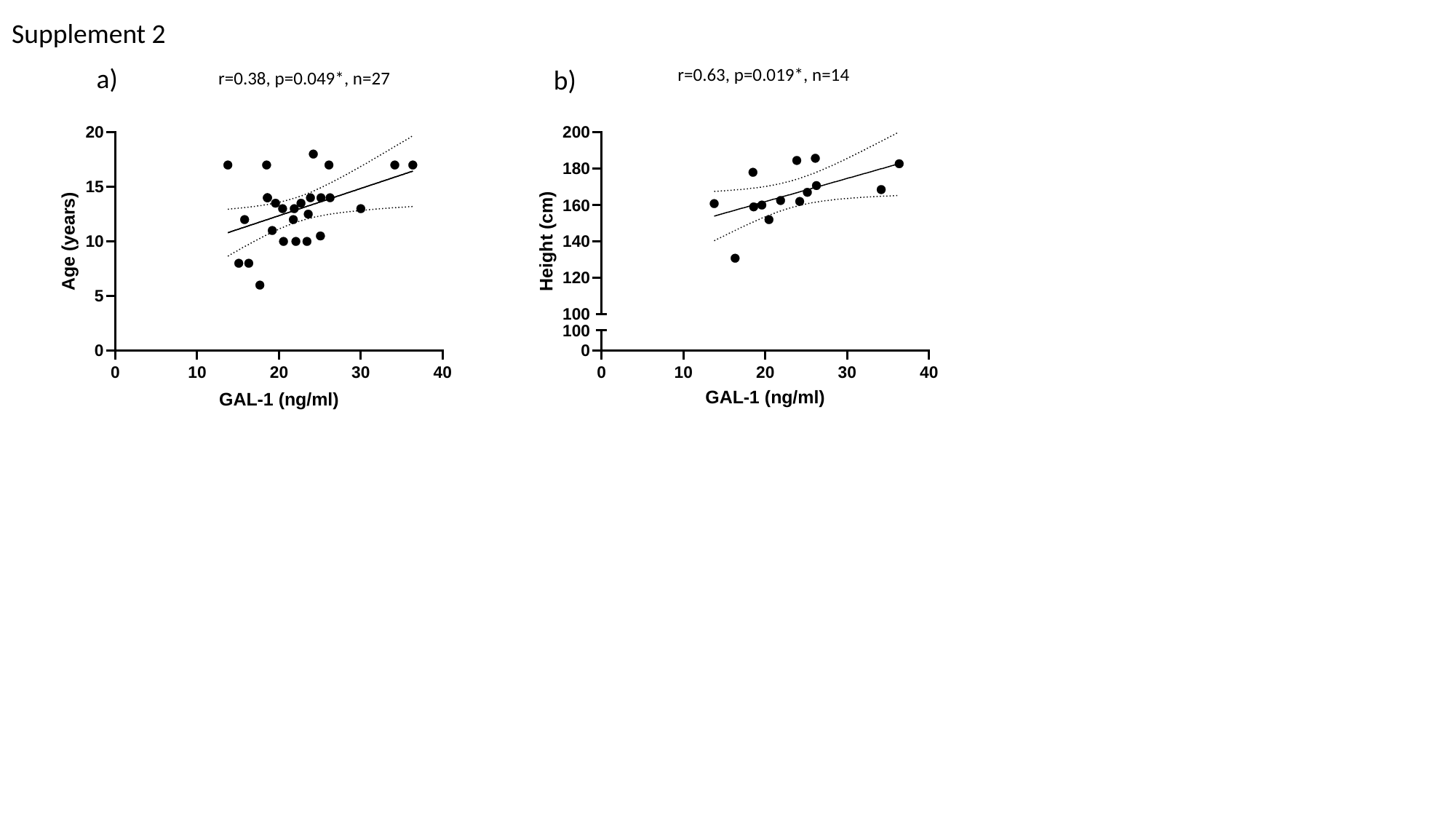

Supplement 2
a)
b)
r=0.63, p=0.019*, n=14
r=0.38, p=0.049*, n=27

Supplement: uxad131_suppl_Supplementary_Materials [file uxad131_suppl_supplementary_materials.zip › uxad131_suppl_Supplementary_Data_S2.pptx]

## Slide 1
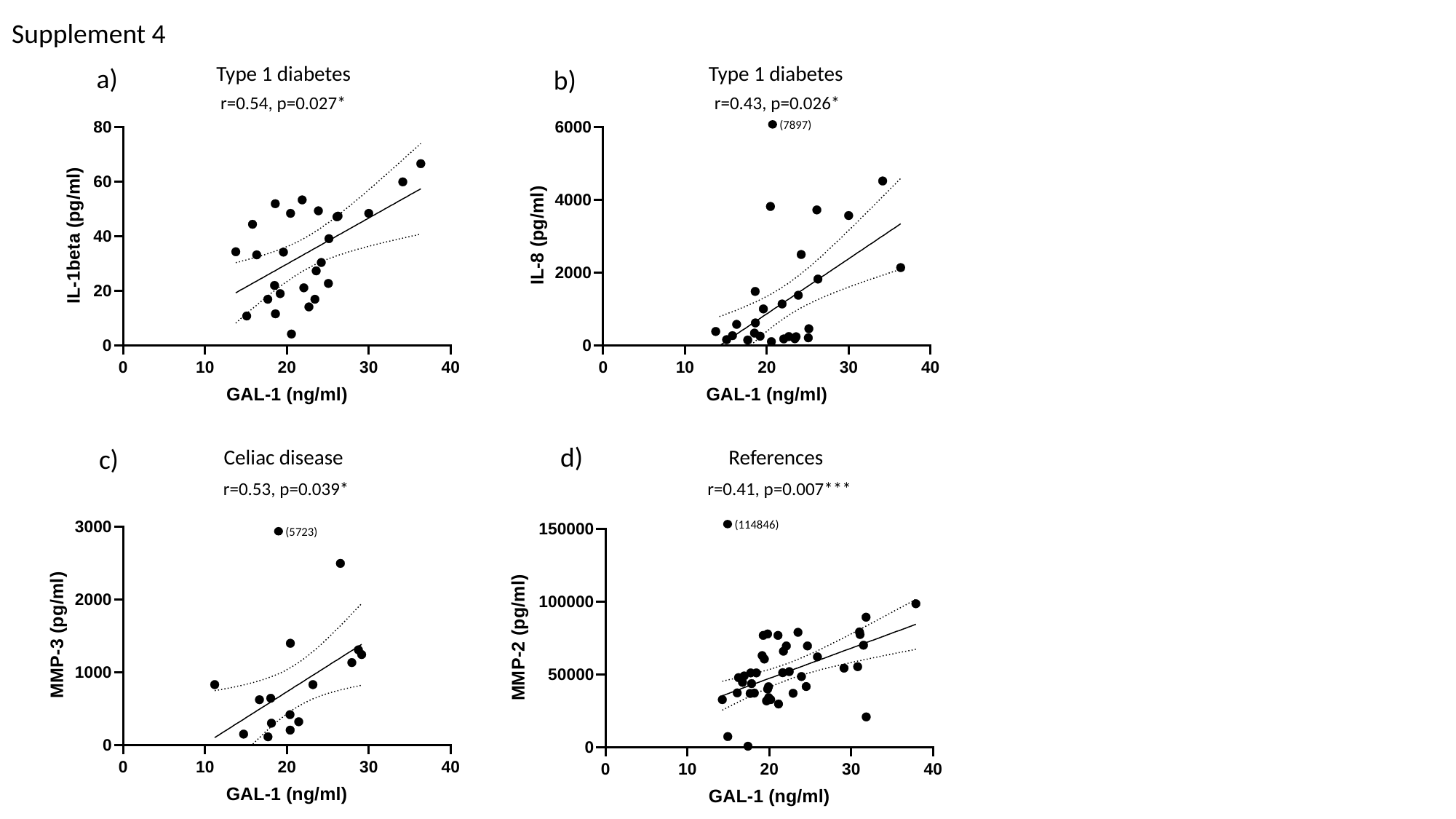

Supplement 4
Type 1 diabetes
Type 1 diabetes
a)
b)
r=0.54, p=0.027*
r=0.43, p=0.026*
(7897)
d)
c)
Celiac disease
References
r=0.53, p=0.039*
r=0.41, p=0.007***
(114846)
(5723)

Supplement: uxad131_suppl_Supplementary_Materials [file uxad131_suppl_supplementary_materials.zip › uxad131_suppl_Supplementary_Data_S4.pptx]
